# Brainshift correction using navigated intraoperative ultrasound informs intraoperative decision-making during glioma surgery

**DOI:** 10.1007/s00701-025-06457-z

**Published:** 2025-04-29

**Authors:** Ashwin Rai, Vikas Singh, Prakash Shetty, Aliasgar V Moiyadi

**Affiliations:** 1 Department of Neurosurgery, Tata Memorial Centre, Mumbai, 400012 India; 2 Department of Health Sciences, Homi Bhabha National Institute, Mumbai, India

**Keywords:** Neuronavigation, Brainshift, Accuracy intraoperative ultrasound, Shift correction

## Abstract

**Background:**

Brainshift can hamper the accuracy of neuronavigation systems in intra-axial tumor surgery. Correction of brainshift becomes imperative to avoid loss of accuracy and erroneous assessment of residual tumor as well as its relationship to critical eloquent substrates.

**Method:**

This paper describes a case of a frontal tumor close to motor cortex. Workflow for rigid image fusion (RIF) based iUS-MR correction of brainshift is demonstrated highlighting its accuracy and clinical value in assessing tumor margins as well as functional boundaries.

**Conclusion:**

iUS-MR fusion provides a cost-effective, accurate and practical solution for observation and correction of brainshift.

**Supplementary Information:**

The online version contains supplementary material available at 10.1007/s00701-025-06457-z.

## Background

Neuronavigation (NN) uses preoperatively acquired MR images to guide intraoperative decisions. Its accuracy can be severely hampered by the phenomenon of brainshift (BS). Brainshift is defined as any factor, physical, surgical, or biological, that violates the rigid body assumption of neuronavigation creating a difference between the reported location of anatomy in the virtual image and patient spaces [3]. Various factors contribute to this [2, 7, 8, 10].

BS is a dynamic process and the assumption in navigation systems that a patient’s head is a rigid body is only valid for the initial stages of the surgical procedure [1]. Physical factors generally produce linear shifts. As tumor resection progresses, tissue loss, fluid loss, presence of pneumocephalus and gravity induced changes lead to complex deformation and elastic shifts which can often be significant. For resection control in intra-axial tumor surgeries, this can lead to loss of accuracy and erroneous assessment of residual tumor as well as its relationship to critical eloquent substrates [5, 9]. 

BS can also be of concern in Deep Brain Stimulation (DBS) procedures, where it can alter the location of the preoperatively defined surgical target and can affect the accuracy of electrode placement or the site of lesioning [4]. Similar difficulties may be encountered for other targeted procedures (biopsy or catheter placements).

BS estimation can done by using direct measurements of physical landmarks on the cortical surface [2]. With the advent of intraoperative MRI (iMRI) and intraoperative ultrasound (iUS) more accurate tools have been employed for this purpose. iMRI helps in imaging updates, evaluation of the extent of tumor resection during surgery, identifying surrounding functional structures to minimize morbidity and compensating for the effect of brain shift. MR-MR fusion algorithms theoretically have the best accuracy for BS correction. However, the logistics of frequent iMRI updates, the need for MRI compatible instruments and the high cost of iMRI makes it less practical [1]. iUS provides a cost-effective and practically efficient option and the intraoperative repeatability of iUS offers considerable benefits. Moreover, the development of 3D navigated ultrasound (3D nUS) has increased the popularity of this technology in recent years [1]. Registration based fusion (RBF) of iUS with pre-operative MRI enables observation of brain shift using fixed landmarks like the septum pellucidum, choroid plexus, falx, and ventricular wall [8]. Since the emergence of navigated iUS as a popular and reliable tool for BS assessment, attempts have been made to use iUS to update RBF [1]. More recently, commercial solutions for such iUS based linear BS correction are available [6]. This is based on a rigid image fusion (RIF) of iUS-MR to calculate and update the default registration-based fusion (RBF), thereby correcting BS. Whereas linear BS is easier to correct, elastic deformations can be very challenging. We describe the application of this technique.

### Case description and relevant anatomy

**Fig. 1:**
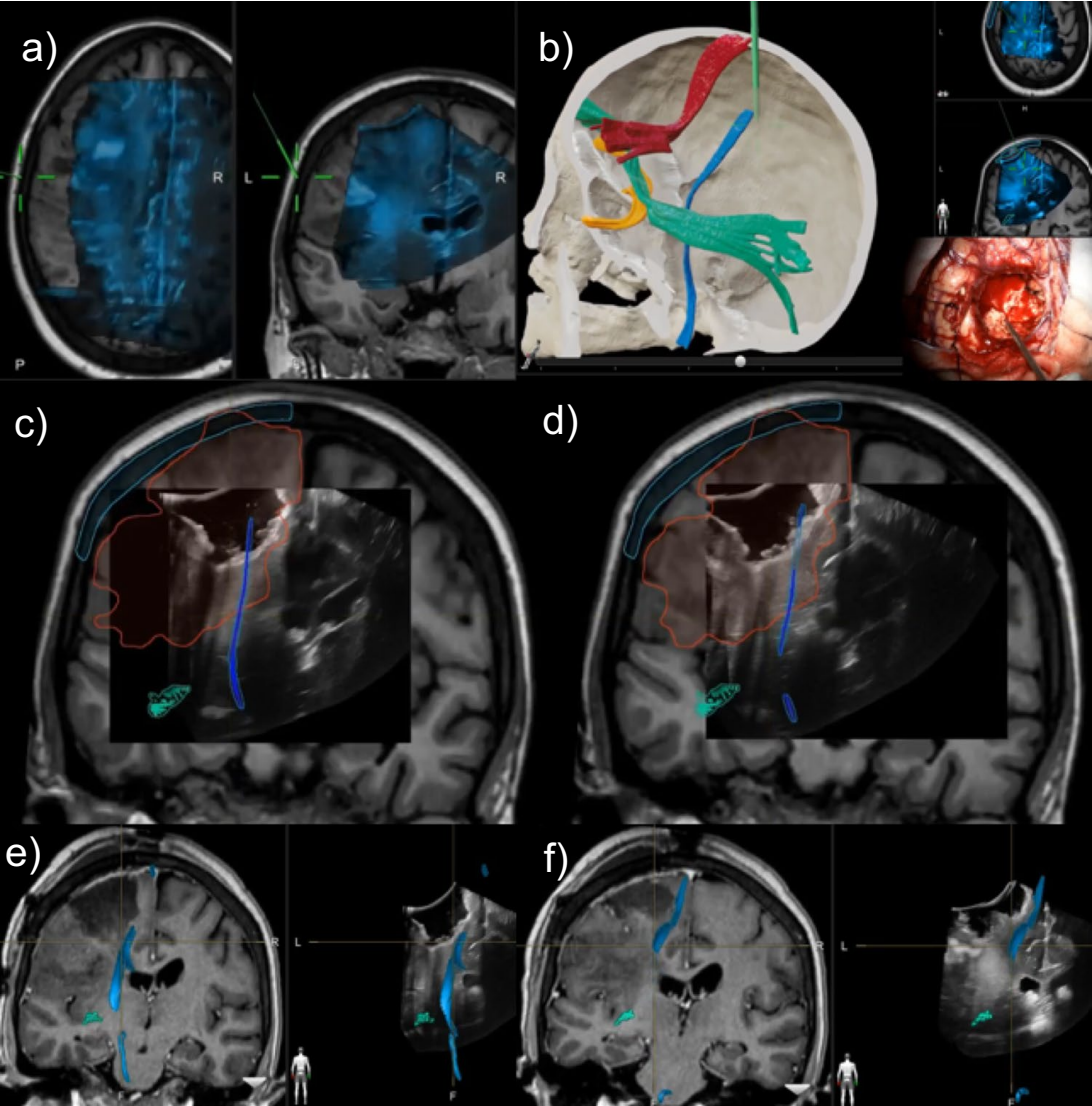
Serial occurrence and correction of brainshift (BS) using navigated ultrasound **a**) Pre-operative fused MRI and iUS with BS **b**) Post resection checking the position of corticospinal tract (in blue color) with pointer in the surgical field and intraoperative ultrasound overlaid on the preoperative MRI **c**) Corticospinal tract seen misaligned in the middle of the resection cavity as depicted by the US in the US-MR fusion images **d**) After application of rigid image fusion and BS correction, the US and MR images are realigned. The corticospinal tract is now seen at the edge of resection cavity which corroborated with the subcortical mapping findings (positive MEPs in the edge of the resection cavity) **e** **& f)** Post op MRI with tractography was fused with post resection iUS showing preserved corticospinal tracts at the tumor resection margin. Note the high correlation of the postoperative MRI-DTI with the shift corrected MR in (**d**)

A 31 year old female presented with right sided hemiparesis and multiple episodes of generalised seizures since 6 months. MRI revealed a left frontal lesion, abutting the motor cortex. Posteriorly it reached the descending corticospinal tract (CST) fibres subcortically. Surgical resection was planned with the aim of maximally debulking the tumor, maintaining a margin from the CST fibres. .Navigated ultrasound (as described below) was used to identify tumor extent and assess residue as well as to account for any brainshift. Subpial resection of the tumor-bearing gyrus was performed. Intraoperatively DTI (Fig. 1) was used to navigate towards the margins of the tumor where the CST were located and this was verified by subcortical dynamic motor mapping. Post resection ultrasound confirmed the location of the CST after applying brainshift correction as described below.

### Description of the technique

**Fig. 2:**
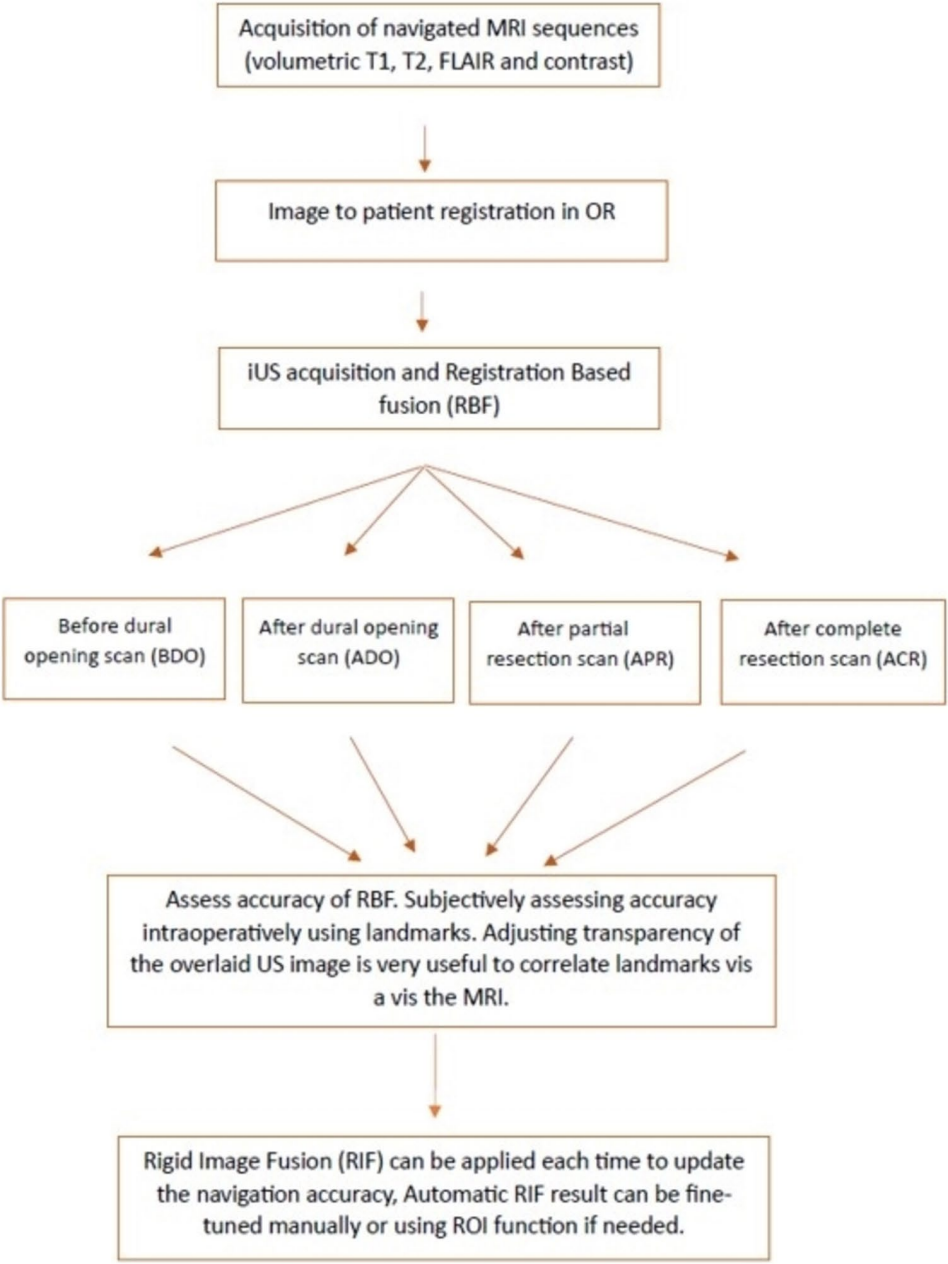
Workflow for Rigid Image fusion-based brain shift correction using intraoperative ultrasound

A commercial system (Kick, Brainlab AG, Germany) was used for neuronavigation (NN). Standard preoperative 1.5T MRI (Ingenia, Philips) was performed and imported into the NN. Tractography (Elements Fibertracking, Brainlab AG, Germany) and semi-automated outlining of the tumor volume and other objects (Elements SmartBrush, Brainlab AG, Germany) was done. Navigated iUS was obtained by using the bk5000 machine (BK Medical Holding Company, GE Healthcare, United States; N13C5 curvilinear probe) digitally integrated with the NN to enable a registration-based fusion (RBF) of iUS and MRI (Ultrasound Navigation, Brainlab AG, Germany). Multiple 3D iUS scans were acquired at different stages throughout the surgery (Before Dural Opening, BDO; After Dural Opening, ADO, and After Completion of Resection, ACR). BS was seen as early as the BDO scan, and again in the ACR scans. Acquisition of a good quality and wide field iUS is imperative. At each stage, a careful qualitative assessment of the fusion accuracy is done and if needed, brainshift correction using RIF (Snap to MRI, Brainlab) was applied. When evaluating the accuracy, adjusting the transparency of the overlaid US image is very useful in gauging the correlation of various landmarks. Similarly, the use of the “spyglass” function in the fusion module helps carefully evaluate the correspondence of the landmarks. The workflow is shown in Fig. 2 and the video (supplementary material 1) describes the technique in an illustrative case in detail. Figure 1 depicts the serial occurrence and correction of brainshift using 3D nUS.

### Indications

All brain tumor surgeries where navigated iUS is used as a localization (biopsy or targeted procedure) or resection control (tumor surgery) tool.

### Advantages

- Can be repeated as often as needed. There can be propagation of the brain shift correction, often reducing the need to apply BS correction in subsequent steps (as in ADO stage in our case) provided no new BS appears.
- Additionally, iUS helps as a gold standard for resection control.
- Correction of the MRI can potentially restore accuracy of all associated information like DTI which is very useful in guiding the functional margins, as in our case, corroborating the functional mapping results.

### Limitations

- Requires a break in the surgical workflow to apply and assess the outcome. Automation in future can overcome this issue.
- At the end of resection, shifts are usually non-linear deformation. Hence, in its present form, this technique may not be adequate in all cases depending on the quantum and nature of the non-linear shifts.

### How to avoid complications

- There are no complications with this technique.
- In order to ensure accuracy, meticulous registration and optimal iUS acquisition is essential.
- Careful attention to the landmarks is required to evaluate brain shift intraoperatively.

## Summary points

- Various physical, biological and surgical factors can lead to intraoperative brain shift affecting the accuracy of NN systems.
- Shifts can be linear (usually due to technical and physical factors) or elastic deformations (due to tumor resection and brain deformations), the latter being more complex.
- Loss of registration accuracy can impact the reliability of image-guided procedures including multimodal image fusion (registration based fusion).
- Correction of brainshift is essential to restore navigational accuracy during surgery.
- Co-registration of iUS with preoperative MRI images can help in accurate measurement as well as real time correction of the brain shift using a rigid image fusion technique.
- iUS is a practical, convenient, and effective option for BS correction.
- Careful navigation registration, and acquisition of a good wide-field, high quality iUS is required to accurately evaluate and correct the brain shift intraoperatively and avoid errors.
- iUS can be repeated and co-registered with preoperative MRI at multiple stages during the surgery which allows for accurate brain shift measurement and correction throughout the surgery.
- iUS also serves as an independent resection control tool during tumor resections.
- Complex non-linear BS are difficult to correct and require more developments in this technology.

## Supplementary Information

Below is the link to the electronic supplementary material.Neurosurgery

ESM1 Video with voiceover shows the application of the RIF based iUS correction of brainshift in a case of right frontal glioma. (MP4 126 MB)

## Data Availability

No datasets were generated or analysed during the current study.
